# Seasonal effect—an overlooked factor in neuroimaging research

**DOI:** 10.1038/s41398-023-02530-2

**Published:** 2023-07-03

**Authors:** Rui Zhang, Ehsan Shokri-Kojori, Nora D. Volkow

**Affiliations:** Laboratory of Neuroimaging, National Institute on Alcohol Abuse and Alcoholism, National Institutes of Health, Bethesda, MD 20892-1013 USA

**Keywords:** Neuroscience, Psychiatric disorders

## Abstract

In neuroimaging research, seasonal effects are often neglected or controlled as confounding factors. However, seasonal fluctuations in mood and behavior have been observed in both psychiatric disorders and healthy participants. There are vast opportunities for neuroimaging studies to understand seasonal variations in brain function. In this study, we used two longitudinal single-subject datasets with weekly measures over more than a year to investigate seasonal effects on intrinsic brain networks. We found that the sensorimotor network displayed a strong seasonal pattern. The sensorimotor network is not only relevant for integrating sensory inputs and coordinating movement, but it also affects emotion regulation and executive function. Therefore, the observed seasonality effects in the sensorimotor network could contribute to seasonal variations in mood and behavior. Genetic analyses revealed seasonal modulation of biological processes and pathways relevant to immune function, RNA metabolism, centrosome separation, and mitochondrial translation that have a significant impact on human physiology and pathology. In addition, we revealed critical factors such as head motion, caffeine use, and scan time that could interfere with seasonal effects and need to be considered in future studies.

## Introduction

Seasonality has been reported for mood and behavior in patients with psychiatric disorders [1] as well as in healthy individuals alongside inter-individual variations [2]. Growing evidence suggests an essential contribution of monoamine neurotransmitters to seasonal fluctuations of psychiatric symptoms. For instance, blunted seasonal dynamics of serotonin reuptake transporter (SERT) availability [3] and cerebral monoamine oxidase A (MAO-A) levels [4] were observed in patients with seasonal affective disorder compared to healthy individuals. Seasonal fluctuations in neurotransmitters are expected to subsequently modulate brain function. In contrast to the various studies on neurotransmitter activity and seasonality, very few studies have investigated seasonal effects on brain functions [5]. A cross-sectional study from Belgium reported that basic attentional processes were associated with day length, whereas higher-level executive brain responses covaried with day-to-day day length variations [6]. Another study from the US showed that the amplitude of P300 event-related brain potential, which reflects processes involved in high-level cognition such as evaluation and decision-making, was larger in young subjects tested in spring/summer than in fall/winter [7, 8].

In the last decade, resting-state functional magnetic resonance imaging (rfMRI) studies have expanded our understanding of brain functional neurocircuitry in a task-free condition [9]. However, no studies have examined seasonal effects on intrinsic brain functional networks. Furthermore, studies that applied longitudinal design to examine seasonal effects are very sparce. Even though some cross-sectional studies had large sample sizes to cover various time points throughout the year, they cannot replace longitudinal studies when investigating intra-individual variations. Here, we leveraged two open-source datasets i.e., MyConnectome [10] and Kirby weekly projects [11] with rfMRI measurements obtained over longer than a 1-year period. We examined associations of resting state functional connectivity (RSFC) with day length and its day-to-day variations. Consistent cross-sectional evidence suggests seasonal fluctuation in the human transcriptome [12–14]. However, longitudinal evidence is still lacking. Thus, we explored seasonal effects on peripheral gene expression using the RNA-sequence data collected in the MyConnectome project. Despite the limitation of case studies in generalizing the results, we hope the knowledge obtained from these single-subject studies can inform neuroimaging studies of critical factors that need to be considered in future studies of seasonality.

## Materials and methods

### Participants and rfMRI imaging acquisition

#### MyConnectome

This is a longitudinal single-subject dataset collected over 1.5 years. The subject was a right-handed Caucasian healthy male aged 45 years at the study onset. In total 84 usable 10-min rfMRI sessions (Figs. S1 and S2) were collected. rfMRI scans were performed at fixed times of the day under eyes-closed conditions (Monday [13 sessions at 5:00 pm], Tuesday [40 sessions at 7:30 am], and Thursday [31 sessions at 7:30 am]). rfMRI data were collected on a Siemens Skyra 3 T MRI scanner using a 32-channel head coil and multiband-EPI sequence (TR/TE = 1160/30 ms; multiband factor = 8; flip angle = 63°; voxel size = 2.4 × 2.4 × 2 mm). For the ethical review of this study please see Poldrack et al. [10].

#### Kirby

The subject was a healthy male aged 40 years at the time of the first scan. In total, there were 156 sessions over a span of 185 weeks [11] (Figs. S3 and S4). Scans were performed weekly typically on Thursdays at 11:30 am and under eyes-closed conditions. The rfMRI data were collected on 3 T Philips Achieva Scanner using a 16-channel neuro-vascular coil and a multi-slice SENSE-EPI pulse sequence with TR/TE = 2000/30 ms, SENSE factor = 2, flip angle = 75°, voxel size = 3 × 3 × 3 mm. The longitudinal single-subject study was performed under protocols approved by the Institutional Review Board at Johns Hopkins University School of Medicine. Signed informed consent was obtained.

### rfMRI preprocessing and analyses

rfMRI data from *MyConnectome project* were preprocessed according to a pipeline developed at Washington University, St Louis [15]. Artifacts were reduced using frame censoring, regression, and spectral filtering. For detailed preprocessing steps please see Poldrack et al. [10]. After preprocessing, the mean time course from each parcel was extracted. For parcellation, we used (1) the individual subject parcellation which consists of a total of 630 regions (616 parcels and 14 subcortical regions) that were assigned to 12 networks [10]; (2) Glasser parcellation (360 ROIs) [16] to test whether the results consisted using a different atlas.

*For Kirby dataset*, MRI data were processed using the minimal preprocessing pipeline of the Human Connectome Project (HCP) [17]. The T1w image was segmented and spatially normalized to the stereotactic space of the Montreal Neurological Institute (MNI) space using routines from the University of Oxford’s Center for Functional Magnetic Resonance Imaging of the Brain (FMRIB) Software Library (FSL) [18], and FreeSurfer (Martinos Center for Biomedical Imaging, Charlestown, Massachusetts, USA). The fMRI data underwent motion correction and normalization to MNI space with 2-mm isotropic resolution. Following surface data extraction, the data were further denoised by regressing out 12 movement regressors (3 rotational, 3 translational, and their derivatives) and censoring (Power FD > 0.25). Twenty-two sessions were excluded because less than 50 frames were left after censoring. The mean time course from each parcel was then extracted using the Glasser atlas [16].

*For both datasets*, Pearson’s correlation coefficients between all pairs of parcels were computed and then converted to normally distributed *Z*-scores using the Fisher transformation for the subsequent analyses. The relationships between head motion (Power FD) [15] and day length, as well as its day-to-day variations, were also examined.

### Blood sample (MyConnectome)

Weekly blood samples (48 blood draws; Fig. S5) enabled us to examine seasonal effects on gene expression. To assess gene expression across the whole genome, transcription profiling was performed on RNA extracted from peripheral blood mononuclear cells. Blood was drawn every Tuesday around 8:00 am immediately after the MRI scan and the subject was fasted and had no caffeine on Tuesdays.

### Gene network analysis (MyConnectome)

We used processed RNA-sequencing data to explore gene expressions associated with day length and its day-to-day variations. Please see Poldrack et al. [10] for methods. To avoid multiple comparisons, we reduced data by using a network approach. First, we applied weighted gene co-expression network analysis (WGCNA) to the RNA-sequencing data (WGCNA R package Version 1.70-3) to cluster genes that co-expressed throughout the year [19, 20]. For data preparation, we regressed against RNA integrity number (RIN) values for each session. For WGCNA analysis, a robust bicorrelation mid-weight estimator was used to estimate correlations. The power for soft thresholding was chosen as 14 based on the scale-free criterion. Modules of highly correlated genes were detected using hierarchical clustering. The first principal component from each module [module eigengenes (ME)] was extracted and its correlations with day length and its day-to-day variations were examined. Benjamini–Hochberg’s false discovery rate (FDR) was used to correct for multiple comparisons [21].

Next, we used DAVID (Version 2021) functional annotation tool, which provides typical gene–term enrichment (overrepresented) analysis, to identify the most relevant (overrepresented) biological terms and pathways associated with genes involved in modules significantly associated with day length and its day-to-day variations [22, 23]. The default background set was used. The annotation analyses included Gene Ontology biological processes (GO-BP), pathways (Reactome [24] and KEGG database [25]), and disease (Genetic Association Database [GAD]) [26]. The significance was determined using the modified Fisher’s exact test [22, 23] and corrected for multiple testing with *P*_FDR_ < 0.05. EnrichmentMap (Version 3.6.6) [27], a Cytoscape (Version 3.10.0) plugin was used for functional enrichment visualization [28].

### Other measurements (MyConnectome)

The PANAS-X was used to assess *the positive* (high energy, concentration, experiencing pleasure) *and negative affect* (distress, unpleasurable engagement) [29] after each scan. Higher scores indicate a *stronger* experience of positive or negative affect. *Total sleep time* (minutes) was measured on most nights before scanning sessions using a *ZEO* sleep monitor. *Self-reported sleep quality* (1 [extremely poor] to 7 [extremely good]) was assessed in the morning. *Naked weight* was measured upon waking using the *FitBit* Aria scale. *The severity of psoriasis* (1 [extremely bad] to 7 [extremely good]), *stress* (1 [extremely bad] to 7 [extremely good]), *total time spent outdoors* (hours), and *consumed alcohol* in standard drinks were assessed by self-reports in the evening [10].

### Statistical analyses

*Daylength and its day-to-day variations* were calculated as the daytime plus civil twilight on the study days using the R package “suncalc”, where calculations were based on the geographic location of the study locations: MyConnectome data (Austin, TX, USA: Latitude =30.286, Longitude = −97.739); Kirby weekly data (Baltimore, Maryland, USA: Latitude = 38.290; Longitude = −76.61). Gain/losses of day length were calculated by subtracting the day length of the day prior to scan day from the day length of the scan day.

We used the lm function in R to examine the effect of day length and its variation on RSFC and included weekdays as a nuisance variable in the linear model. Additionally, to examine whether caffeine/food consumption modulated the seasonal effect in the MyConnectome dataset, we separately tested the day-length effect on Tuesday (no caffeine/fasted) and Thursday (fed/caffeinated) using Spearman’s rank correlation. A significance level was set at a connection-level FDR-corrected *p* < 0.05.

Pearson’s correlations were used to assess correlations between day length, day-to-day day length variations, and other measures including positive and negative affect, sleep, stress, weight, psoriasis severity, time spent outdoors, and alcohol consumption in the MyConnectome dataset. If normality was violated i.e., Kolmogorov–Smirnov test was significant, a non-parametric Spearman’s rank correlation was performed.

## Results

### Resting-state functional connectivity

**Fig. 1:**
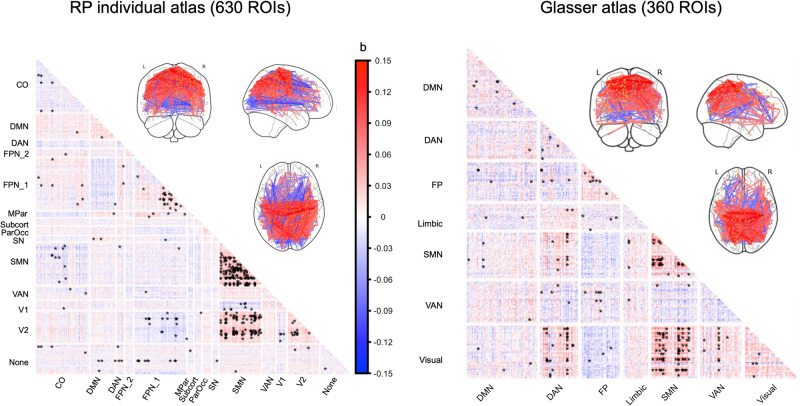
RSFC associated with day length (MyConnectome). Relationship between day length and RSFC after regressing out weekdays. Regression coefficients b are plotted. Significant FC (*P*_BH_ < 0.05) are marked with *. Left: Parcellation using subject-specific atlas; Right: Parcellation using Glasser atlas and assigned to Yeo 7 networks.

**Fig. 2:**
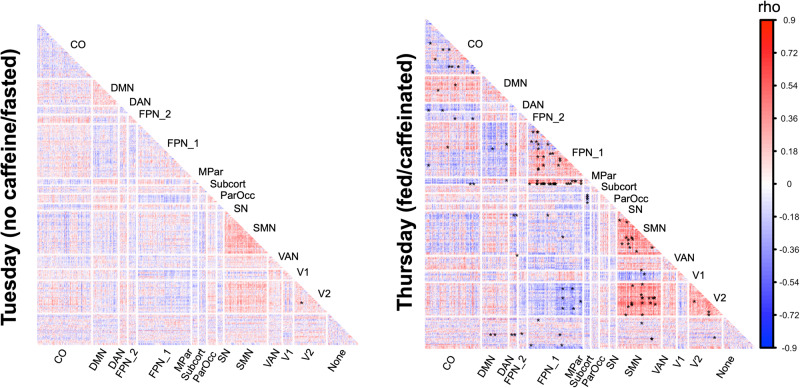
Caffeine enhanced seasonal effect on RSFC (MyConnectome). Correlations (rho) between day length and RSFC for Tuesday (no caffeine/fasted) and Thursday (caffeinated/fed) are plotted separately. Significant FC (*P*_BH_ < 0.05) are marked with *. BH Benjamini–Hochberg correction for multiple testing. Module labels: none: unassigned, DMN default mode network, V2 second visual network, FP1 primary frontoparietal network, V1 primary visual network, DA dorsal attention network, VA ventral attention network, SN salience network, CO cingulo-opercular network, SMN somatomotor network, FP2 secondary frontoparietal network, MPar medial parietal network, ParOcc parieto-occipital network, subcort subcortical regions.

For the MyConnectome project, using a different atlas, the RSFC findings consistently showed that day length was most prominently associated with greater functional connectivity within the sensorimotor network (SMN) and between the SMN and the visual network (Fig. 1). However, no significant RSFC associations were found with day-to-day variations in day length. In this dataset, the subject was an fMRI expert, and the head motion was minimal: Framewise displacement (FD) ranged from 0.04 to 0.06. Head motion was not associated with day length (rho = −0.158, *p* = 0.150) or its variations (rho = 0.159, *p* = 0.149) even when only including the morning scans on Tuesday and Thursday (71 sessions) and excluding the evening scans on Monday (13 sessions) to lessen the circadian variability in the data (day length rho = −0.139, *p* = 0.247; day length variation: rho = 0.095, *p* = 0.559). Further, caffeine/food enhanced the seasonal effects on RSFC such that the effect of day length was only significant on Thursday with caffeine/food consumption but not on Tuesday without caffeine/food intake before scans (Fig. 2 and Table S1).

**Fig. 3:**
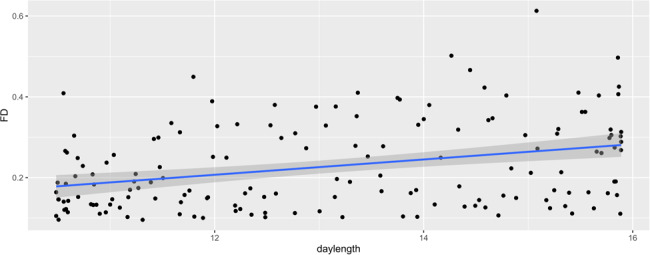
Seasonal effect on head motion (Kirby). Head motion during fMRI scans (FD) was positively associated with day length.

For the Kirby weekly project, we did not find RSFC significantly associated with daylength or daylength variations. However, in this dataset, a head motion was greater compared to the MyConnectome project: FD ranged from 0.10 to 0.61. Notably, a head motion was positively associated with day length (rho = 0.316, *p* < 0.001) (Fig. 3) but not day length variations (rho = 0.156, *p* = 0.052).

### Gene expression

Using WGCNA, 35 co-expression modules were identified (See Table S2 for the hub genes of each module). Five of them (modules 1, 3, 7, 16, and 25) were positively associated with day length while 10 of them (modules 2, 4, 5, 11, 12, 13, 17, 30, 32, and 33) were negatively associated with day length (all |*r*| > 0.36, *P*_BH_ < 0.05; Fig. S6) but not with day-to-day daylength variation (all |*r*| < 0.28, *p* > 0.056).

**Fig. 4:**
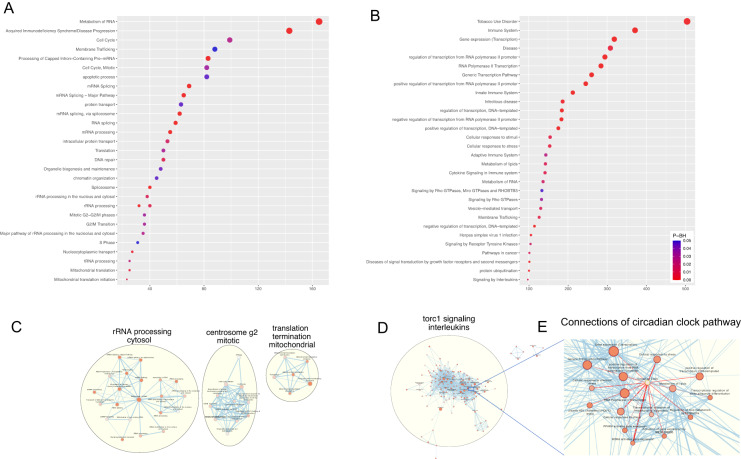
Biological processes, pathways, or disease associated with day length. The results of enrichment analyses for genes that were (**A**, **C** Positively; **B**, **D**, **E** Negatively) associated with day length were represented. **A**, **B** The top 30 enriched terms with the greatest gene count is shown. The color represents the adjusted *p*-value of the enrichment scores (BH: Benjamini–Hochberg correction), while the dot size and *x*-axis represent the number of gene counts. **C**–**E** Gene-set similarity mapping: edges represent the overlap between gene sets with a cutoff value of 0.5. Gene sets with similar functional themes was clustered. **E** Overlapping of the circadian pathway with other biological processes and pathways.

Since genes within a module were not necessarily biologically related, we pooled genes from modules positively and negatively associated with day length respectively for functional annotation using DAVID. Genes involved in rRNA processing in the cytosol, centrosome separation, and mitochondrial translation increased their expression with longer day length (Fig. 4A, C and Table S3). Genes that were downregulated with longer day length were ones strongly relevant to immune function (Fig. 4B, D and Table S4). Genes enriched in the circadian pathway were also downregulated and involved in other biological processes and pathways that regulate immune functions (Fig. 4E).

### Other measures

Longer day length and day-to-day day length variation was associated with greater weight (day length: rho = 0.515, *p* < 0.001; day length variation: rho = 0.366, *p* < 0.001). No significant associations were found for mood, sleep, time spent outdoors, stress, alcohol consumption, or severity of psoriasis.

## Discussion

The MyConnectome data analyses showed that longer day length (but not day-to-day day length variation) was associated with RSFC within the SMN and between the SMN and the visual network. This is consistent with a preclinical study showing seasonal effects on plasticity in the somatosensory cortex [30]. Moreover, a recent human study, reported that resting-state fMRI signal variance dropped endogenously (i.e., not evoked by external cues) at times coinciding with dawn and dusk, notably in sensory cortices including the bilateral visual, somatosensory, and right auditory cortices [31]. In the MyConnectome dataset, 71 rfMRI scans were performed at the same time in the morning (i.e., 7:30 am on Tuesday and Thursday). The time between scans and dawn, which was associated with day length (rho = 0.908, *p* < 0.001), varied across the seasons (range from −33.58 to 87.5 min). In contrast to the summer scans, in winter the dawn was later in the day such that the scan time was about or prior to dawn (Fig. S7). The SMN exhibits strong recurrent connections consistent with localized processing of external stimuli [32]. Therefore, SMN could be a network that conveys information about dawn and dusk, which vary throughout the year, to the rest of the brain. The sequent question is whether seasonal variation in SMN results from the subject’s light exposure prior to the scan (e.g., during travel to the research center) indicating a reactive brain response; or reflects anticipatory adaptions of spontaneous neural activity at twilight in preparation for expected environmental changes, indicating a proactive response. A cross-sectional study under strictly controlled laboratory conditions demonstrated seasonality of cognitive brain responses in healthy participants after living without any seasonal cues for 4.5 days suggesting that there might be a “photic memory” for the photoperiod to which the participants were exposed prior to the study [6]. If the SMN association with day length reflects a proactive brain response, SMN might receive information from upstream regions that signal the timing of twilight such as the entrainment of the internal clock by light [33].

Another important question is whether there is an interplay between the time of day and seasonal effects. In other words, whether scan time affects the observed seasonal effect, for instance, whether the seasonal effect would be smaller or observed in other regions when subjects are scanned in the middle of the day rather than near dawn or dusk. The subject in the Kirby weekly project gave us some insight into this since the subjects were scanned weekly typically on Thursdays at 11:30 am under eyes-closed conditions. We did not find RSFC significantly associated with daylength or daylength variations. However, longer day length (but not day length variation) was associated with greater head motion, which could reflect worse inhibitory control [34, 35]. Thus, in this dataset, head motion interfered with the results, and the post-hoc statistical control for head motion could have attenuated the seasonal effect confounding the findings i.e., whether the negative results reflected a lack of seasonal effect on RSFC when the subject was tested at midday or reflected the regressing out of the neurobiological factors that underly head motion and are also influenced by day length.

In the MyConnectome project, we did not find changes in mood, sleep, stress, outdoor activities, or alcohol consumption across seasons, although weight increased with longer day length and greater day length variations. Inter-individual differences in SMN adaptations to external seasonal changes might account for resilience to seasonal variations in mood and behavior since patients with seasonal affective disorders had reduced seasonality at the molecular level that could affect RSFC [3, 4]. Comparing seasonal effects on intrinsic brain networks between healthy participants and patients with psychiatric disorders would be a critical next step. There have already been some observations for associations of brain network dynamics with different affective states. In bipolar disorder, the shift of depressive and manic phases has been suggested to relate to the balance between default mode network (DMN) and SMN [36, 37].

We further compared seasonal effects on days with and without caffeine/food consumption. Interestingly, caffeine/food enhanced the seasonal effect on RSFC. Clinical [38, 39] and preclinical studies [40] suggest that caffeine modulates monoamine transmitters that display seasonal fluctuations. Since caffeine is the most widely used stimulant worldwide, it should be systematically examined by future studies.

Additionally, our findings were in line with previous studies supporting a strong seasonal effect on immune function [12, 13]. Interestingly, we also found that the expression of genes involved in circadian pathways was downregulated alongside longer day length. These circadian genes engaged in other biological processes and pathways that support immune functions. Research on the interaction between the immune system, circadian rhythms, and brain function is a very promising area for studying seasonal effects [41–44]. In contrast, genes involved in processes of RNA metabolism, centrosome separation, and mitochondrial translation were upregulated with longer day lengths. Disruptions of these processes are relevant to human physiology and are associated with various diseases including cancer, cardiovascular diseases, nervous system diseases, and infections [45–47].

## Conclusions

We took advantage of databases from two single-subject studies and documented seasonality in functional brain networks, particularly in SMN. Single-subject projects have revealed important factors that could influence the sensitivity to seasonal effects such as head motion, caffeine use, and time of day of the scan. Genetic findings revealed biological pathways including the neuroimmune pathway that might contribute to the seasonal effect on brain network dynamics and the involvement of circadian modulation. Large sample sizes are needed for additional investigations. Since a stronger seasonality has been associated with more severe phenotypes in psychiatric disorders [1], further investigation of seasonality in RSFC in patients with psychiatric disorders might be clinically valuable to monitor and personalize treatments. So far, seasonal effects are understudied and often controlled as a covariate in brain research, although seasonal changes are one of the most prominent changes in the environment. Future endeavors that take advantage of neuroimaging, metabolomics, and sleep and circadian measures are needed to advance our knowledge of biological processes underlying seasonal adaptations.

## Supplementary information

Supporting information

Table S3 Functional annotation of genes positively associated with day length

Table S4 Functional annotation of genes negatively associated with day length

## Data Availability

All data files are available under websites http://myconnectome.org/wp/data-sharing/ (MyConnectome) and https://www.nitrc.org/projects/kirbyweekly/ (Kriby).
