# Supplementary material for: Seasonal effect—an overlooked factor in neuroimaging research
Source: Transl Psychiatry. 2023 Jul 3;13:238. doi: 10.1038/s41398-023-02530-2 (PMC10318079; doi:10.1038/s41398-023-02530-2)
Supplement: Supplementary file 1 — Supporting information [file 41398_2023_2530_MOESM1_ESM.docx]

**Supporting Information**

**Seasonal effect- An overlooked factor in neuroimaging research**

Rui Zhang ^a,*^, Ehsan Shokri-Kojori ^a^, Nora D. Volkow ^a,*^

*^a^ Laboratory of Neuroimaging, National Institute on Alcohol Abuse and Alcoholism, National Institutes of Health, Bethesda, MD 20892-1013, USA*

***Corresponding authors:**

Rui Zhang

[rui.zhang@nih.gov](mailto:rui.zhang@nih.gov)

phone: +1(301)402-5861

Nora D. Volkow

[nvolkow@nida.nih.gov](mailto:nvolkow@nida.nih.gov)

Laboratory of Neuroimaging

National Institute on Alcohol Abuse and Alcoholism

National Institutes of Health

10 Center Drive, Room B2L124: MSC 1013

Bethesda, MD 20892-1013, USA

**Figure S1 Photoperiod of days when rfMRI scans were performed (MyConnectome)**

**Figure S2 Day-to-day daylength gain/loss (minutes) (MyConnectome)**

**Figure S3 Photoperiod of days when rfMRI scans were performed (Kirby)**

**Figure S4 Day-to-day daylength gain/loss (minutes) (Kirby)**

**Figure S5 Photoperiod of days when blood samples were collected (MyConnectome)**

**A**

**B**

**Figure S6 Modules associated with daylength**

**A.** Variations of MEs throughout the year. Red: Modules show upregulated gene expression with longer daylength; Blue: Modules show downregulated gene expression with longer daylength **B.** Mean MEs are first calculated for upregulated and downregulated modules respectively. Correlations between mean MEs and daylength are plotted.

**Figure S7 Minutes between scan time (7:30 AM) and dawn for 71 morning sessions (MyConnectome)**

**Table S1 Correlations (rho) between daylength and RSFC on Thursday**

| **ROI1_nr** | **ROI2_nr** | **ROI1_net** | **ROI2_net** | **Rho** |
| --- | --- | --- | --- | --- |
| 595 | 147 | Frontoparietal_1 | Medial_Parietal | 0.81 |
| 461 | 273 | Medial_Parietal | Frontoparietal_1 | 0.80 |
| 595 | 457 | Frontoparietal_1 | Medial_Parietal | 0.79 |
| 340 | 80 | Zero | Dorsal_Attention | 0.79 |
| 461 | 296 | Medial_Parietal | Frontoparietal_1 | 0.78 |
| 519 | 147 | Frontoparietal_1 | Medial_Parietal | 0.78 |
| 461 | 7 | Medial_Parietal | Frontoparietal_1 | 0.78 |
| 461 | 155 | Medial_Parietal | Frontoparietal_1 | 0.76 |
| 332 | 5 | Somatomotor | Visual_2 | 0.76 |
| 213 | 57 | Frontoparietal_1 | Frontoparietal_1 | 0.76 |
| 158 | 156 | Frontoparietal_1 | Frontoparietal_1 | 0.75 |
| 296 | 158 | Frontoparietal_1 | Frontoparietal_1 | 0.75 |
| 457 | 192 | Medial_Parietal | Frontoparietal_1 | 0.75 |
| 522 | 158 | Frontoparietal_1 | Frontoparietal_1 | 0.75 |
| 354 | 72 | Somatomotor | Somatomotor | 0.75 |
| 235 | 158 | Frontoparietal_1 | Frontoparietal_1 | 0.75 |
| 573 | 332 | Visual_2 | Somatomotor | 0.75 |
| 158 | 75 | Frontoparietal_1 | Frontoparietal_1 | 0.74 |
| 534 | 376 | Zero | Somatomotor | 0.74 |
| 449 | 70 | Visual_2 | Somatomotor | 0.74 |
| 51 | 8 | Somatomotor | Somatomotor | 0.74 |
| 455 | 70 | Visual_2 | Somatomotor | 0.74 |
| 461 | 186 | Medial_Parietal | Frontoparietal_1 | 0.73 |
| 467 | 381 | Frontoparietal_1 | Frontoparietal_1 | 0.73 |
| 614 | 318 | Ventral_Attention | Somatomotor | 0.73 |
| 461 | 192 | Medial_Parietal | Frontoparietal_1 | 0.73 |
| 158 | 157 | Frontoparietal_1 | Frontoparietal_1 | 0.73 |
| 519 | 158 | Frontoparietal_1 | Frontoparietal_1 | 0.73 |
| 573 | 49 | Visual_2 | Somatomotor | 0.72 |
| 457 | 190 | Medial_Parietal | Frontoparietal_1 | 0.72 |
| 235 | 213 | Frontoparietal_1 | Frontoparietal_1 | 0.72 |
| 463 | 381 | Frontoparietal_1 | Frontoparietal_1 | 0.72 |
| 428 | 84 | Somatomotor | Somatomotor | 0.72 |
| 285 | 210 | Frontoparietal_1 | Cingulo_opercular | 0.72 |
| 461 | 217 | Medial_Parietal | Frontoparietal_1 | 0.72 |
| 332 | 256 | Somatomotor | Visual_2 | 0.72 |
| 373 | 38 | Somatomotor | Somatomotor | 0.72 |
| 457 | 296 | Medial_Parietal | Frontoparietal_1 | 0.72 |
| 383 | 64 | Somatomotor | Somatomotor | 0.72 |
| 449 | 332 | Visual_2 | Somatomotor | 0.72 |
| 467 | 457 | Frontoparietal_1 | Medial_Parietal | 0.72 |
| 350 | 70 | Somatomotor | Somatomotor | 0.71 |
| 461 | 285 | Medial_Parietal | Frontoparietal_1 | 0.71 |
| 371 | 259 | Somatomotor | Visual_2 | 0.71 |
| 504 | 364 | Frontoparietal_1 | Frontoparietal_1 | 0.71 |
| 457 | 197 | Medial_Parietal | Frontoparietal_1 | 0.71 |
| 502 | 457 | Frontoparietal_1 | Medial_Parietal | 0.71 |
| 318 | 231 | Somatomotor | Visual_2 | 0.71 |
| 72 | 15 | Somatomotor | Visual_2 | 0.71 |
| 373 | 60 | Somatomotor | Somatomotor | 0.71 |
| 416 | 104 | Cingulo_opercular | Cingulo_opercular | 0.71 |
| 340 | 194 | Zero | Dorsal_Attention | 0.71 |
| 534 | 377 | Zero | Somatomotor | 0.71 |
| 60 | 52 | Somatomotor | Somatomotor | 0.71 |
| 325 | 295 | Zero | Frontoparietal | 0.71 |
| 156 | 147 | Frontoparietal_1 | Medial_Parietal | 0.71 |
| 454 | 388 | Visual_2 | Somatomotor | 0.71 |
| 384 | 259 | Somatomotor | Visual_2 | 0.71 |
| 461 | 298 | Medial_Parietal | Frontoparietal_1 | 0.70 |
| 573 | 448 | Visual_2 | Visual_2 | 0.70 |
| 346 | 223 | Cingulo_opercular | DMN | 0.70 |
| 350 | 51 | Somatomotor | Somatomotor | 0.70 |
| 457 | 156 | Medial_Parietal | Frontoparietal_1 | 0.70 |
| 381 | 340 | Frontoparietal_1 | Zero | 0.70 |
| 461 | 291 | Medial_Parietal | Frontoparietal_1 | 0.70 |
| 474 | 60 | Visual_2 | Somatomotor | 0.70 |
| 381 | 235 | Frontoparietal_1 | Frontoparietal_1 | 0.70 |
| 477 | 457 | Frontoparietal_1 | Medial_Parietal | 0.70 |
| 461 | 235 | Medial_Parietal | Frontoparietal_1 | 0.70 |
| 383 | 377 | Somatomotor | Somatomotor | 0.69 |
| 496 | 494 | Somatomotor | Dorsal_Attention | 0.69 |
| 454 | 70 | Visual_2 | Somatomotor | 0.69 |
| 507 | 147 | Frontoparietal_1 | Medial_Parietal | 0.69 |
| 384 | 256 | Somatomotor | Visual_2 | 0.69 |
| 439 | 157 | Frontoparietal_1 | Frontoparietal_1 | 0.69 |
| 504 | 192 | Frontoparietal_1 | Frontoparietal_1 | 0.69 |
| 213 | 145 | Frontoparietal_1 | Frontoparietal_1 | 0.69 |
| 297 | 114 | DMN | Cingulo_opercular | 0.69 |
| 439 | 192 | Frontoparietal_1 | Frontoparietal_1 | 0.69 |
| 367 | 283 | Frontoparietal_1 | Frontoparietal_1 | 0.69 |
| 379 | 259 | Somatomotor | Visual_2 | 0.69 |
| 594 | 30 | DMN | Medial_Parietal | 0.69 |
| 355 | 60 | Somatomotor | Somatomotor | 0.69 |
| 332 | 259 | Somatomotor | Visual_2 | 0.69 |
| 559 | 448 | Visual_2 | Visual_2 | 0.69 |
| 449 | 374 | Visual_2 | Somatomotor | 0.69 |
| 324 | 141 | Visual_2 | Visual_2 | 0.69 |
| 522 | 147 | Frontoparietal_1 | Medial_Parietal | 0.69 |
| 461 | 288 | Medial_Parietal | Frontoparietal_1 | 0.69 |
| 479 | 461 | Cingulo_opercular | Medial_Parietal | -0.69 |
| 296 | 271 | Frontoparietal_1 | DMN | -0.69 |
| 266 | 147 | Parieto_occipital | Medial_Parietal | -0.69 |
| 147 | 12 | Medial_Parietal | Parieto_occipital | -0.69 |
| 459 | 330 | Cingulo_opercular | Cingulo_opercular | -0.69 |
| 439 | 348 | Frontoparietal_1 | Somatomotor | -0.69 |
| 587 | 459 | Frontoparietal | Cingulo_opercular | -0.69 |
| 508 | 142 | Frontoparietal_1 | Visual_2 | -0.69 |
| 523 | 481 | Zero | Visual_2 | -0.70 |
| 346 | 169 | Cingulo_opercular | Cingulo_opercular | -0.70 |
| 158 | 133 | Frontoparietal_1 | Zero | -0.70 |
| 492 | 36 | Dorsal_Attention | Somatomotor | -0.70 |
| 439 | 142 | Frontoparietal_1 | Visual_2 | -0.70 |
| 351 | 247 | Somatomotor | Visual_1 | -0.70 |
| 213 | 36 | Frontoparietal_1 | Somatomotor | -0.70 |
| 340 | 222 | Zero | DMN | -0.70 |
| 481 | 439 | Visual_2 | Frontoparietal_1 | -0.70 |
| 492 | 149 | Dorsal_Attention | Cingulo_opercular | -0.71 |
| 578 | 27 | Zero | Frontoparietal_1 | -0.71 |
| 461 | 437 | Medial_Parietal | Cingulo_opercular | -0.71 |
| 439 | 259 | Frontoparietal_1 | Visual_2 | -0.71 |
| 512 | 459 | Cingulo_opercular | Cingulo_opercular | -0.71 |
| 340 | 286 | Zero | DMN | -0.71 |
| 459 | 293 | Cingulo_opercular | Cingulo_opercular | -0.71 |
| 504 | 44 | Frontoparietal_1 | Cingulo_opercular | -0.71 |
| 578 | 395 | Zero | Frontoparietal_1 | -0.72 |
| 593 | 324 | Frontoparietal_1 | Visual_2 | -0.72 |
| 331 | 215 | Somatomotor | Zero | -0.73 |
| 594 | 235 | DMN | Frontoparietal_1 | -0.73 |
| 587 | 345 | Frontoparietal | Cingulo_opercular | -0.73 |
| 459 | 393 | Cingulo_opercular | Cingulo_opercular | -0.73 |
| 147 | 139 | Medial_Parietal | Parieto_occipital | -0.74 |
| 533 | 511 | Visual_2 | Frontoparietal_1 | -0.74 |
| 161 | 36 | Dorsal_Attention | Somatomotor | -0.75 |
| 346 | 293 | Cingulo_opercular | Cingulo_opercular | -0.76 |
| 492 | 44 | Dorsal_Attention | Cingulo_opercular | -0.77 |
| 586 | 459 | Cingulo_opercular | Cingulo_opercular | -0.77 |
| 293 | 69 | Cingulo_opercular | Cingulo_opercular | -0.80 |
| 577 | 147 | Parieto_occipital | Medial_Parietal | -0.82 |

**Table S2 Hub genes of 35 coexpression modules identified using WGCNA**

| Mod | gene1 | gene2 | gene3 | gene4 | gene5 | gene6 | gene7 | gene8 | gene9 | gene10 |
| --- | --- | --- | --- | --- | --- | --- | --- | --- | --- | --- |
| 1 | TTC1 | TBCA | PDIA6 | TAF12 | RTFDC1 | DNAJC8 | C19orf43 | RBMX2 | SNRPD3 | EIF2S2 |
| 2 | HECTD4 | IGF1R | RALGAPA2 | MKL2 | ZNF592 | AAK1 | SEC16A | TRRAP | IQSEC1 | ARHGAP35 |
| 3 | FCHSD1 | ARFGAP1 | ACAP1 | SH2B1 | C11orf21 | AARS2 | HGS | STK25 | RHOT2 | TRMT1 |
| 4 | ABCC1 | ZER1 | MAP2K3 | RNPEPL1 | MAST3 | SUFU | WDTC1 | NUP210 | SSH1 | SKI |
| 5 | EIF4E3 | YTHDF3 | MSL2 | AIDA | CCDC6 | GLTSCR1L | PPP3CB | UBQLN2 | RUNX2 | PUM2 |
| 6 | VBP1 | SRP9 | USMG5 | PSMA2 | SCAMP1 | PPP2R3C | ATG5 | STK17A | PSMA5 | PPA2 |
| 7 | RBM5 | SP140 | IK | SLTM | FAM192A | ZC3H15 | LTV1 | EIF1AD | TLK2 | SART3 |
| 8 | CCDC88B | AP5Z1 | SPPL2B | RHPN1 | CROCC | SPSB3 | TRMT2A | MIB2 | SGSM3 | TMC6 |
| 9 | CD226 | ABCC4 | PGRMC1 | TNFSF4 | RAB11A | SMIM3 | DAPP1 | PDE5A | PRTFDC1 | ANXA3 |
| 10 | ATXN2L | PRRC2A | SYNGAP1 | PRRC2B | CAMTA2 | FMNL1 | MNT | MLXIP | MAU2 | SRCAP |
| 11 | CSNK1A1 | ZDHHC2 | FOXN2 | DLAT | PRKCI | FAM126A | SP4 | ERO1L | DNAJC3 | RBM18 |
| 12 | CD46 | RAB3GAP2 | NPAT | DOCK11 | QSER1 | PDCD6IP | FAR1 | EDEM3 | DNAJC13 | CERKL |
| 13 | PIKFYVE | UBR3 | TAF9B | BIRC6 | ZNF292 | MKLN1 | ZNF800 | FAM160B1 | FOXJ3 | MON2 |
| 14 | NBEAL2 | AKNA | SIN3B | ZNF687 | RNF44 | ULK1 | FBXO41 | DOT1L | FAM65A | CRTC1 |
| 15 | BMP6 | EHD3 | JAM3 | LTBP1 | GNAZ | ABCC3 | TSPAN33 | CTDSPL | PTGS1 | TTC7B |
| 16 | MIAT | AHSA2 | DCAF8 | PNISR | VAMP1 | ANKZF1 | SERINC4 | PATL2 | ZBTB40 | STX2 |
| 17 | SCIMP | PLXNC1 | TLR7 | CHST15 | RIN2 | TLR8 | PLBD1 | PPT1 | FAM198B | S100A11 |
| 18 | APBA3 | CYBA | SSBP4 | SIPA1 | NR1H2 | NAPRT1 | TSPO | MBD3 | APRT | SIVA1 |
| 19 | TAPBP | G6PD | ARHGAP1 | SH3KBP1 | AP1B1 | NDRG1 | EIF4G1 | ZYX | ANXA11 | TMEM127 |
| 20 | SLC16A3 | CCDC85B | COPE | YIF1B | FKBP8 | C19orf24 | UBXN6 | SSNA1 | GSDMD | BOP1 |
| 21 | MYLK | PDGFA | TUBA8 | PARD3 | C15orf26 | YWHAH | SLFN14 | RDH11 | SYTL4 | PLCH1 |
| 22 | LRRC25 | ALDH3B1 | PLEKHO2 | RXRA | GNAI2 | KIAA0930 | CD14 | MAN2B1 | CEBPA | CTSB |
| 23 | APLF | CDYL | CCDC175 | CNTLN | SSX2IP | ZCCHC17 | STRN3 | ASPH | CETN2 | X7.Sep |
| 24 | SENP7 | IRAK4 | RICTOR | DDX26B | CCNT2 | KRR1 | PTCD3 | SP3 | ZNF600 | KATNBL1 |
| 25 | TXLNG2P | BTAF1 | CCDC14 | TTC14 | CREBZF | AMY2B | ZNF83 | FAM76B | ANAPC4 | ZDHHC6 |
| 26 | MIOS | DNAJB14 | ZDHHC17 | DHX15 | FAM76B | ZDHHC21 | ZNF678 | IFT80 | SRSF10 | EED |
| 27 | SBF1 | PKD1 | INTS1 | CC2D1A | GAK | SIGIRR | TMEM8A | ADRBK1 | NCLN | NSMF |
| 28 | HMGN4 | SPAST | TMX1 | MDH1 | SELT | PSMD10 | NXT2 | GALNT1 | EID1 | OSTC |
| 29 | TAF1 | SMARCC2 | SRRM1 | RBBP6 | YLPM1 | SETD5 | TNRC6C | BOD1L1 | ZNF318 | MDC1 |
| 30 | ACTN1 | MPL | PEAR1 | CMIP | ANKRD33B | AFAP1 | MGAT4B | VSIG2 | MAML3 | GNAO1 |
| 31 | ERGIC1 | CFL1 | PPP1R11 | PPP1R18 | KIAA2013 | PIGS | DAXX | RALBP1 | OPA3 | CALCOCO2 |
| 32 | ATP6V1A | CPVL | PTEN | CREG1 | HNMT | TNFSF13B | IFNGR2 | IFNGR1 | SCARB2 | ACSL4 |
| 33 | LRRFIP1 | GLG1 | NCOA6 | DIAPH1 | GPATCH8 | SYNRG | IL2RG | AP2B1 | EPB41L2 | EIF5B |
| 34 | PHF3 | TPR | CEP290 | C10orf118 | AKAP9 | PCM1 | PCF11 | ANKRD26 | SMC6 | PRPF40A |
| 35 | FGFR1OP2 | TNFAIP8 | AFTPH | TFAM | CHMP2B | KRCC1 | GTPBP10 | SNX6 | ACTR6 | SH2D1A |
